# Cerebellar contribution to feedforward control of locomotion

**DOI:** 10.3389/fnhum.2014.00475

**Published:** 2014-06-25

**Authors:** Iolanda Pisotta, Marco Molinari

**Affiliations:** Neurological Rehabilitation Department A and CaRMA Lab, I.R.C.C.S. Fondazione Santa Lucia RomeRome, Italy

**Keywords:** locomotion, corticocerebellar loops, feedforward control, movement prediction, sequencing hypothesis

## Abstract

The cerebellum is an important contributor to feedforward control mechanisms of the central nervous system, and sequencing—the process that allows spatial and temporal relationships between events to be recognized—has been implicated as the fundamental cerebellar mode of operation. By adopting such a mode and because cerebellar activity patterns are sensitive to a variety of sensorimotor-related tasks, the cerebellum is believed to support motor and cognitive functions that are encoded in the frontal and parietal lobes of the cerebral cortex. In this model, the cerebellum is hypothesized to make predictions about the consequences of a motor or cognitive command that originates from the cortex to prepare the entire system to cope with ongoing changes. In this framework, cerebellar predictive mechanisms for locomotion are addressed, focusing on sensorial and motoric sequencing. The hypothesis that sequence recognition is the mechanism by which the cerebellum functions in gait control is presented and discussed.

## Introduction

Based on animals and humans studies, much has been learned about how the cerebellum coordinates normal movement and how it contributes to motor adaptation and motor learning.

Cerebellar damage does not cause a loss of movement; instead, it effects clear and consistent abnormalities in movement, including lack of coordination, increased variability, tremor, and poor accuracy. Notably, cerebellar damage induces greater impairments to movements that require predictive control versus those that require reactive control. As demonstrated by Morton and Bastian (2006) using an elegant task that was based on splitbelt treadmill walking, cerebellar damage impairs the ability to adapt to predictable but not sudden unpredictable changes. Recently developed functional theories on predictive control of the cerebellum explain the effects of cerebellar damage on eye and limb movements and on walking (Koziol et al., 2014).

In motor control theories, the term “predictive” refers to the feedforward component of a movement that is planned in advance and is unchanged by online peripheral feedback. Predictive control is typically assessed at the earliest stage of the movement, during which corrections that are based on peripheral feedback are not possible. This type of control is used to make any online corrections that might be necessary as a movement unfolds, and it requires that the conditions of later movement stages be known in advance. Although the hypothesis that the cerebellum is important for predictive control is not new, claims of its importance in locomotive control are relatively recent (Morton and Bastian, 2006). Further, current data on the significance of cerebellar predictive control in areas outside of the motor domain and for supporting learning and functional recovery have piqued the interest of neuroscientists in the hopes of better understanding cerebellar control mechanisms.

In this framework, we will discuss cerebellar predictive mechanisms for locomotion, focusing on the type of information that is processed—sensorial or motoric—and on sequence recognition as the mechanisms for understanding cerebellar function in making predictions.

## Cerebellum and feedforward control

The cerebellum has an exquisitely simple cellular organization, which has been well known since the beginning of the last century, based on the work of Ramon y Cajal (see Sotelo, 2008). Since then, scientists have been intrigued by its function. Nevertheless, after more than a century of dedicated studies, there is no consensus on how the cerebellum operates. Among the various theories that exist, the hypothesis that the cerebellum mediates predictive motor control in locomotion is gaining momentum (Bastian, 2006).

The forward model of motor control postulates that to make a motor-to-somatosensory prediction, the cerebellum receives an efference copy of a motor command from the primary motor cortex. This information allows the cerebellum to make a prediction with regard to the sensory consequences of such motor commands, allowing the musculoskeletal system to prepare to successfully execute a movement. During movement, predicted sensations are then compared with the actual incoming sensations. If there is a positive match, the pattern is maintained for the next movement. The lack of a match is associated with an alert signal that is sent back to the motor cortical and subcortical areas, which activates feedback movement corrections and calibration of the forward model (Shadmehr et al., 2010). This process allows the corticocerebellar circuit to act as somatic event detectors that respond, particularly to unexpected stimuli (Restuccia et al., 2007). Various studies on the internal forward model have demonstrated that the cerebellum generates motor-to-somatosensory predictions (Izawa et al., 2012; Popa et al., 2012; Knolle et al., 2013).

Thus, consensus is building that the cerebellum is more involved in learning to associate motor commands with novel sensory consequences–i.e., the forward model—than in learning to correlate sensory goals with novel motor commands (the inverse model).

## Sequencing and prediction

In 1997, Braitenberg, Heck, and Sultan proposed sequence detection and generation as the basic operational mode of the cerebellum in the motor domain (Braitenberg et al., 1997). Since then, the sequencing properties of cerebellar processing have been studied extensively, and sequencing has been reported to be the more frequently impaired function in a large cohort of subjects with cerebellar damage (Tedesco et al., 2011).

**Figure 1:**
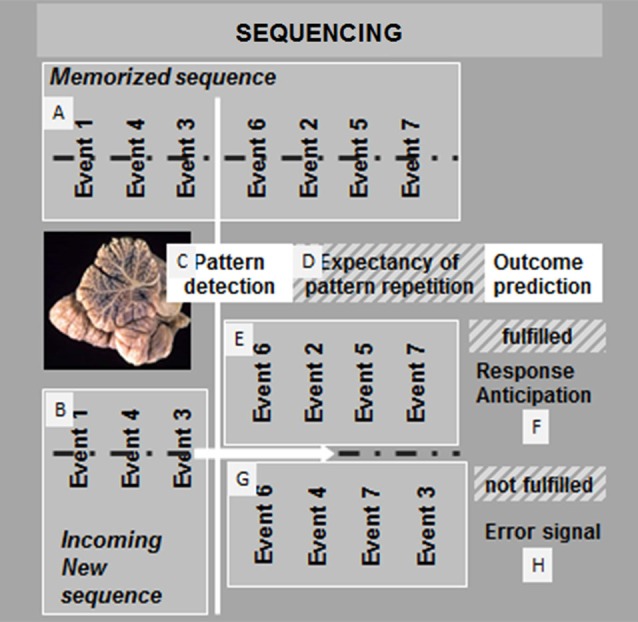
**Proposed mechanism of cerebellar sequencing for prediction**. Incoming events are continuously monitored in the cerebellar circuits. Relations between events are compared in the cerebellar corticonuclear microcomplex (Ito, 2005) and stored in a working memory buffer **(A)**. Through the same mechanisms, sequences of new incoming events are compared with previously stored event sequences **(B)**. If a match is recognized **(C)**, then an expectancy of repetition is generated **(D)**. The cerebellar monitoring of the flow of events continues, and as long as the prediction is maintained **(E)**, response anticipation is conveyed, and feedforward control can function smoothly **(F)**. If prediction fails **(G)**, then an error signal is activated by the cerebellar output system **(H)**, and feedforward control is interrupted or corrected.

Few years ago, we proposed to consider sequencing the basic mechanism that allows cerebellar prediction in all functional domains (Molinari et al., 2008). Traditionally, sequencing has not been recognized as a discrete cognitive function, and it can be defined as a supramodal function, the relationships with other functions of which, such as working memory and timing, remain unknown. Acquiring and acting on a serial order of events is a fundamental ability that effects sequencing structure knowledge. To recognize that stimuli are presented in a particular order, sensory information that pertains to a stimulus must be kept active in a working memory system and compared with subsequent stimuli. Like many instrumental abilities, sequence knowledge can be acquired incidentally through experience (implicit learning) or intentionally through explicit effort (declarative learning). A schematic of cerebellar sequence mechanisms for prediction is shown in Figure 1.

Whenever feedforward control is needed, the cerebellum intervenes by identifying predictable patterns of motor or cognitive command sequences and linking them with learned sensory or cognitive consequences. This process allows anticipatory responses to be generated in all relevant cerebellar domains.

Cerebellar input has a facilitating effect on the contralateral cerebral cortex, and chronic cerebellar damage (Di Lazzaro et al., 1994) and cerebellar conditioning transcranial magnetic stimulation (TMS; Grimaldi et al., 2014) reduce the excitability of the contralateral motor cortex. Furthermore, the cerebellar influence on the cerebral cortex is not limited to motor areas, and the nature and functional significance of the overall cerebellar influence over the cerebral cortex is the subject of much debate (Dalal et al., 2013). At least for the motor domain, it is widely accepted that cerebellar input conveys information for sensory motor integration.

TMS experiments in rats support this model (Ben Taib et al., 2005; Oulad Ben Taib and Manto, 2008). In rats, as in humans, the enhancement of excitability in the contralateral motor cortex after sustained somatosensory stimulation is cerebellum-dependent (Kaelin-Lang et al., 2002; Luft et al., 2002). Cerebellum-dependent neurophysiological changes are not limited to the motor cortex, and cerebellar output might also directly affect the somatosensory cortex. The parietal cortex projects to the cerebellum through the pontine nuclei in a topographically organized manner (Schmahmann and Pandya, 1989), and it receives the cerebellar return loop through the thalamus (Giannetti and Molinari, 2002; Allen et al., 2005; Clower et al., 2005). Cerebellar influences on the parietal somatosensory cortex have been demonstrated in cats (Kolodziejak et al., 2000) and in patients with unilateral cerebellar lesions (Restuccia et al., 2001). In subjects with unilateral cerebellar damage, late N24 and P24 components of the somatosensory-evoked potentials decline significantly in the contralateral somatosensory cortex (Restuccia et al., 2001). A magnetoencephalography (MEG) study that compared expected and unexpected sensory stimuli in evoking cerebellar and cortical responses has linked the somatosensonsory evoked potentials findings with the prediction and sequencing theories of cerebellar function. A regular train of somatosensory stimuli induces evoked potentials in the contralateral somatosensory cortex and ipsilateral cerebellum. If the stimulus is omitted at random, while no activity is recorded in the somatosensory cortex, cerebellar activity is markedly enhanced (Tesche and Karhu, 2000). This response after an unpredictable omission in a predictable sequence has been interpreted as proof of the ability of the cerebellum to code expectancy (Ivry, 2000).

Clinical evidence of cerebellar function in coding expectancy has been confirmed by 2 groups, both of which used mismatch negativity protocols. Restuccia et al. (2007) used a somatosensory mismatch negativity (S-MMN) protocol in patients with unilateral cerebellar lesions. S-MMN is believed to be generated by differences between current and prior inputs, supported by an automatic change-detection cortical process. This process is blocked or impaired if the cerebellar input is absent. Subjects who are affected by unilateral hemispheric cerebellar stroke do not develop S-MMN responses in the contralateral cortex. Similar findings have been observed in an MMN auditory paradigm (Moberget et al., 2008), focusing on timing expectancy. Both studies have demonstrated that the cerebellum is part of the MMN circuit and that it is critical for generating expectancy and making sensory predictions.

These findings complement the theories on cerebellar function in the prediction of sensory events (Nixon, 2003) and the longstanding hypothesis that the cerebellum acts as a comparator (Ito, 2005). In this theoretical framework, it is conceivable that through a comparison of time and space characteristics of actual and preceding stimuli, predictable event sequences can be recognized and stored. Thus, sequencing in the sensorimotor domain is evident, but is this also true when cognitive functions are considered?

Behavioral or script sequencing can be defined as the process that allows spatial and temporal relations to be recognized correctly among behaviorally relevant actions (Sirigu et al., 1998). Script sequencing is altered in subjects with cerebellar damage who are tested in an ad hoc card sorting task (Leggio et al., 2011) and is interpreted as a prediction deficit in the cognitive/behavioral domain. Card sequencing tasks require visual or verbal material to be examined to understand spatial, temporal, and/or semantic relationships and correctly reconstruct the strings in logical sequences.

The test in Leggio et al. (2008) consisted of 11 sets of cards, each comprising 6 cartoon-like drawings, including sentences (to examine verbal factors), behavioral figures (for behavioral factors), and abstract figures (for spatial factors), that were to be ordered in a logical sequence by patients. The influence of the lesion was analyzed by grouping patients by lesion type (focal or atrophic) and lesion side (right or left). Patients with cerebellar damage developed cognitive sequencing impairments, and lesion side and characteristics of the material that were to be sequenced correlated. Specifically, patients with left lesions performed poorly only on script sequences that were based on pictorial material, and patients with right lesions encountered difficulties with script sequences that required verbal elaboration. The presence of right/left and pictorial/verbal differences is consistent with the hypothesis that cerebrocerebellar interactions are organized in segregated corticocerebellar loops, in which specificity is related to the characteristics of the information that is processed—not to the mode of function (Leggio et al., 2008).

## Cerebellar gait

Locomotion can be considered a purposeful, goal-directed behavior that is initiated by signals that arise from volitional processing in the cerebral cortex or emotional processing in the limbic system and sustained by basic locomotor motor patterns that are generated by spinal interneuronal networks—i.e., the central pattern generator (CPG) circuits. Locomotor control mechanisms are complex and, in addition to the CPGs, involve various subcortical and cortical control areas (for review, see Takakusaki, 2013). In this network, the cerebellum is considered dispensable for steady-state locomotion but crucial for avoiding obstacles and adapting to novel conditions.

Cerebellar gait ataxia is characterized by staggering, irregular stepping, veering, and excessive high lifting of the feet above the ground. This clinical condition has been linked to the inability to control relative movements between leg joints during locomotion. Starting from clinical observation, the coordination of multijoint activity through the scaling of movement variables has been considered the core of cerebellar motor function (Topka et al., 1998). Of the movement variables that are cerebellum-dependent, the timing of muscle activity, especially of antagonist muscles, has long been favored (Frysinger et al., 1984).

In 2001, Earhart and Bastian questioned cerebellar function in the timing or scaling of individual joint movements during gait by asking subjects with cerebellar damage to step on a surface that was inclined at various angles while walking. Healthy subjects mastered the task by using several temporal strategies, with systematic shifts in the timing of muscle activity and peak joint angles, based on the changes in inclination. Notably, cerebellar subjects were able to produce appropriate timing shifts at most joints, demonstrating preserved selection of the basic timing of motor patterns. Conversely, the presence of abnormal relative joint movements and the decomposition of movement implicated the cerebellum in adjusting the relative movement of multiple joints, especially to accommodate external constraints (Earhart and Bastian, 2001). Collectively, animal studies and clinical evidence have demonstrated cerebellar function in adaptive gait control, effecting constant recalibration of walking patterns to navigate various terrains and environments smoothly.

Cerebellar adaptation is not based on sensory feedback information. Subjects with cerebellar damage are impaired in locomotor tasks that require prediction, whereas they have good control when reactive control is needed (Morton and Bastian, 2006). As discussed, one possible mechanism of sustaining prediction is sequencing, which can intervene at various levels of locomotor control. Thus, similar to what has been observed in sMMN paradigms (Restuccia et al., 2007), fixed sequences of sensory information, funneled by spinocerebellar fibers during locomotion (Jankowska et al., 2011), have been hypothesized to be recognized by the cerebellum, effecting correct prediction of the neuromuscular requirements of the subsequent step. Alterations in the predicted sequence will enhance the cerebellar output system, allowing cortical and brainstem locomotor regions to adapt.

Conversely, subjects with cerebellar atrophies are not only impaired in managing obstacles and adapting to novel environment, they develop ataxic gait in well-learned environments and on smooth surfaces (Mari et al., 2014), implicating cerebellar processing in controlling steady-state locomotion. This clinical profile also exists in subjects with cerebellar stroke; nevertheless, gait ataxia is generally mild, from which patients recover well (Bultmann et al., 2014). Pascual-Leone and colleagues (Farzan et al., 2013) recently reported that 21 days of cerebellar TMS reduces gait ataxia in patients who are affected by idiopathic late-onset cerebellar atrophy. This finding suggests that low-frequency TMS reduces the inhibitory control of the cerebellar cortex over the dentate nucleus, favoring the function of dentate nucleus output.

To this end, we would like to advance an alternative hypothesis. Considering the established function role of motor learning and adaptation in allowing forward control strategies to be generated and the lack of cerebellar influence on reactive adjustments and well-learned automatic movements, it is conceivable that cerebellar TMS inhibits the cerebellar output, allowing motor circuits to act in the absence of cerebellar influences. Thus, the experimental condition that was proposed by Pasqual-Leone and colleagues could approximate a cerebellar focal lesion—i.e., after a stroke. Both conditions are associated with better locomotion than in the presence of cerebellar atrophy.

It follows that cerebellar gait ataxia due to cerebellar atrophy might be the result of erroneous cerebellar predictions. Altered cerebellar processing will insert virtual errors into the forward control models, inducing continuous correction of the ongoing motor command. If this hypothesis is true, the inhibition of cerebellar processing—e.g., by TMS or tDCS—would improve gait in subjects with cerebellar ataxia but will have little or no effect on subjects with ataxia due to focal cerebellar damage. Conversely, focal damage to the cerebellum is associated with balance and gait problems, primarily in the acute/subacute phase followed by efficient spontaneous functional recovery (Bultmann et al., 2014). This evidence suggests that the motor system recovers more efficiently from the absence of cerebellar processing than from alterations to it. This view is supported by recent literature on cerebellar stimulation (Farzan et al., 2013; Ferrucci et al., 2013; Grimaldi et al., 2014), suggesting that transcranial cerebellar stimulation is a feasible neurorehabilitation intervention that can be used to treat gait ataxia (Block and Celnik, 2012).

## Conclusion

The importance of feedforward control in motor control and the significance of cerebellar processing in this function are well established. Nevertheless, in locomotion control, cerebellar function is neglected, and studies have focused primarily on spinal and cortical locomotion control mechanisms. Current evidence implicates forward models as more important locomotion control mechanisms, but the relative importance of forward and inverse models to locomotion remains unknown. Recent reports indicates that cerebellar processing intervenes in locomotion by providing advance information on subsequent step events, suggesting how such motor prediction can be obtained per the sequencing hypothesis of cerebellar function. In nearly all cerebellar functional domains—from motor to cognition—cerebellar symptoms can be attributed to impairments in recognizing repeated sequenced patterns. Only recognition of a previously experienced pattern allows a prediction to be made and thus effective feed-forward control to be instigated. This theory of cerebellar function has implications for the symptomatic treatment of gait disturbances, and preliminary results on magnetic and electrical modulation of cerebellar function are guiding the development of an effective treatment for ataxic gait.

## Conflict of interest statement

The authors declare that the research was conducted in the absence of any commercial or financial relationships that could be construed as a potential conflict of interest.
